# Toward Audit-Ready Standards for Synthetic Cohorts in Health Decision Science

**DOI:** 10.34133/hds.0414

**Published:** 2026-01-23

**Authors:** Mulavagili Vijayasimha

**Affiliations:** Department of Medical Laboratory Technology, University Institute of Allied Health Sciences, Chandigarh University, Mohali 140413, Punjab, India.

Dear Editor,

The recent article by Ferreira et al. [1] on GPT-4-generated population samples and their alignment with real psychometric data makes an important contribution to current discussions on synthetic datasets in health decision science. Their findings highlight a larger issue: as synthetic cohorts increasingly influence trial simulation, economic modeling, and regulatory evaluation, the field still lacks audit-ready standards to ensure transparency, reproducibility, and decision-grade reliability.

Recent analyses of generative systems reinforce that validation is the most underdeveloped component in the synthetic-data pipeline. As de Hond and colleagues [2] emphasize, frameworks originally designed for predictive models cannot simply be transplanted to generative large language models used for synthetic data creation. Instead, synthetic cohorts require a distinct validation logic that evaluates distributional fidelity, extremal-case behavior, fairness across subgroups, and uncertainty propagation—elements rarely reported in current practice.

Work on foundation models further demonstrates that data provenance, sampling strategies, and model-specific biases markedly influence downstream clinical or analytical outputs [3]. Without transparent documentation of these steps, the reproducibility of synthetic cohorts and their suitability for health-policy use become difficult to audit.

Another critical area is privacy risk, which persists even when datasets appear high-fidelity or well-sanitized. Giuffrè and Shung [4] show that privacy protection and utility are not guaranteed simply because the data are synthetic. Likewise, membership-inference studies continue to reveal vulnerabilities in synthetic datasets when not rigorously stress-tested [5]. These findings underscore the need for standard practices that mandate explicit evaluation of privacy risks before synthetic cohorts are deployed in real decision environments.

Given the pace of adoption of synthetic datasets in digital health, regulatory science, and economic modeling, I propose the development of Audit-Ready Synthetic Cohort Standards comprising

• structured validation reports covering fidelity metrics, fairness, out-of-distribution behavior, robustness, and uncertainty characterization
• transparent provenance logs, including prompts, model versions, dataset sources, preprocessing steps, and sampling strategies
• decision-context fitness assessments ensuring that synthetic cohorts meet minimum quality thresholds aligned to the analytical or policy context
• adversarial and stress-testing protocols to evaluate privacy leakage, membership-inference susceptibility, and structural vulnerabilities
• mandatory reporting guidelines for journals, requiring detailed documentation in methods and supplementary materials

Such standards would strengthen scientific credibility, protect patient interests, and accelerate regulatory preparedness. Ferreira et al.’s contribution [1] is an important catalyst, and I hope that this letter advances discussion toward a unified audit-ready framework for safe, transparent, and decision-fit synthetic health datasets.
